# Extension of Lecture Terms in Medical Colleges

**Published:** 1848-10

**Authors:** 


*Extension of Lecture Terms in Medical Colleges.*—The editor of the Medical Examiner corrects a misapprehension into which we, and others, had fallen, with respect to the action of the American Association at the meeting in May last, upon the subject of extending lecture terms in medical colleges. We had been led to suppose (and it appears to have been a current impression) that, at the meeting mentioned, the association receded from its former recommendation of extending to six months, and recommended an extension to only five months. This, however, is an error. The resolution relating to this subject reported to the association and adopted, read thus:—

"*Resolved*, That this committee reiterate and strongly recommend to the Association, a practical observance of the Resolutions appended to the Reports of the Committees on Preliminary Education, and on the requisites for graduation, submitted to the Medical Convention which assembled in Philadelphia in May, 1847."

